# Development of a Lebanese food exchange system based on frequently consumed Eastern Mediterranean traditional dishes and Arabic sweets

**DOI:** 10.12688/f1000research.27461.1

**Published:** 2021-01-11

**Authors:** Maha Hoteit, Edwina Zoghbi, Alissar Rady, Iman Shankiti, Ayoub Al-Jawaldeh

**Affiliations:** 1 Faculty of Public Health, Lebanese University, Beirut, Lebanon; 2 World Health Organization Lebanon Country Office, World Health Organization, Beirut, Lebanon; 3 World Health Organization Regional Office for the Eastern Mediterranean, World Health Organization, Cairo, Egypt

**Keywords:** Exchange list, carbohydrate, protein, fat, traditional dishes, Lebanon

## Abstract

**Background**: The important role of Mediterranean diet was elucidated in various clinical and epidemiological studies underlying its impact on reducing the burden of non-communicable diseases in Mediterranean and non-Mediterranean populations.

**Objective**: The aim of this study was to convert the recipes of the Lebanese traditional dishes into meal planning exchange lists whose items are expressed in grams and adjusted to Lebanese household measures (cups and spoons) that could be used by healthcare professionals.

**Methodology**: Thirty traditional Lebanese dishes were collected in which the carbohydrate, fat and protein were analyzed using Association of Official Analytical Chemists procedures then followed by a calculation of exchange lists of foods per serving using Wheeler method.

**Results**:  The variations in macronutrients and fiber content were found among the Lebanese dishes. Carbohydrate was lowest (1.1g/100g) and protein was highest (29.7g/100g) in *Shawarma Dajaj* whereas fat content ranged between 0.5 and 22.4 g/100 g in the dishes. For each dish and according to each serving size, carbohydrate, milk (whole milk, reduced fat or skim), fat and protein (lean meat, medium fat meat and high fat meat) exchanges were calculated.

**Conclusion:** This study provides healthcare professionals, dietitians and consumers the chance to proficiently plan traditional-type dishes, ensuring prominent dietetic and medical nutritional therapy practices and patient's self-control.

## Background

The Eastern Mediterranean Region (EMR) is currently facing rapid social and economic changes, urbanization, and advances in technology along with a shift in the prevalence of non-communicable diseases (NCDs) due to the obesogenic environment, nutrition transition and modification of lifestyle patterns characterized by changes in food intake and a reduction in physical activity attitudes and practices ^1^. Lebanon is an Arab country with a population of over six million ^2^ and is considered as part of the EMR. Few data have discussed food attitudes and practices in Lebanon; however, recent evidence published in 2019 from two surveys enrolled previously between 1997 and 2008/2009, showed a shift in the Lebanese diet with regards to an expansion in energy intake from 1728 ± 24 kcal in 1997 to 1877 ± 15 kcal in 2008/2009 and dietary fat from 34.63 ± 0.32% in 1997 to 36.97 ± 0.21% in 2008/2009, coupled with decreases in carbohydrate (CHO) (48.97 ± 0.23% in 2008/2009 compared to 51.32 ± 0.36% in 1997), in fruit consumption (4.72 ± 0.15% vs. 7.36 ± 0.22% in 1997) and a decrease in micronutrient dietary density along with a decrease in the consumption of milk (1.09 ± 0.08% vs. 1.53 ± 0.11% in 2008/2009 and 1997, respectively). No changes were reported with regards to protein and fiber intake ^3^. According to the World Health Organization (WHO) statistics, the worldwide probability of premature death due to NCDs accounts for 70% of the 41 million deaths each year. In Lebanon in 2016, 91% (10,334 individuals) of all deaths were due to NCDs ^4^. Furthermore, among the 10,334 premature deaths from NCDs, 27.2% of deaths were due to cancer. Around 31% of the population was obese, 13% had diabetes type II, 20% had raised blood pressure and 36% had a sedentary lifestyle ^4^. 

The implementation of food composition tables and development of exchange lists is drawing attention at a national and international level due to the recommendations and guidelines ^5^ published by the public and private sectors with the purpose of implementing programs aiming to ameliorate medical nutritional therapeutics for widely distributed NCDs. Food composition tables and exchange lists also have an impact on marketing, on the trade of products and on consumer safety and health ^6^. Many factors influence meal planning for a healthy diet, of which food choices, personal preferences, ethnic behaviors and tradition may increase the responsibility and commitment of health care professionals and dietitians in promoting improved nutrition at an individual and community level, through adherence to food composition tables and exchange lists ^7^.

At the Middle Eastern level, there is a lack in regional and national exchange lists that incorporate traditional foods. The food exchange list ^8^, which helps in translating recipes into food serving sizes and energy intake, was designed to assist individuals in an easily operated and easily understood way in improving their healthy eating habits and in adhering to a healthy diet plan. In the food exchange list, foods available in the same category can be used reciprocally without any change in the quantities of macronutrients and energy yielded by a dish. According to numerous sources, there is a need to add cultural relevance to food exchange lists to improve NCD management ^9^, taking into consideration ethnic variations and traditions which may have enormous influence on individual and community health ^10^. Thus, the key success factor for improving health care professionals, dietitians and individuals’ adherence to healthy food choices is to design and implement culturally accepted exchange lists that add traditional dishes into their meal planning ^7^. Currently, Lebanese dietitians are using the American Dietetic Association exchange list to design meal plans. However, this exchange list is limited by a gap in traditional meals consumed in Lebanon. In spite of the availability of a few national food composition tables that involve a limited number of dishes common in Lebanese cuisine ^11, 12^, ingredients and preparation methods differed substantially across time and these are outdated. The aim of this study is to enable Lebanese healthcare professionals and dietitians to develop, design, and implement practical culture-based meal plans that include traditional cooking.

## Methods

### Dish/sweet selection

The definition of ‘composite dishes’ is “dishes consumed at main meals (i.e. lunch or dinner), whose preparation involves culinary skills and contains ingredients from at least three of five main food groups: meat/poultry/fish and eggs; dairy products; fruits and vegetables; starchy foods including legumes; added sweets and fats” ^13^. The list of Lebanese composite dishes frequently consumed by Lebanese citizens was retrieved from a study performed in 2005 on a representative sample of 799 Lebanese adults ^13^, and in line with a study conducted in 2009 where the objective was to compare the consumption of traditional dishes between Lebanon and France ^14^. As for Arabic sweets, a broad selection of almost all types frequently consumed by Lebanese people was compiled. The Lebanese diet includes a range of foods with often complex recipes, and it is rarely possible to analyze all of the types of dishes. In such cases, laboratory analysis of the traditional dishes and a calculation of some nutrients should be achieved. The names of the dishes and Arabic sweets most eaten by Lebanese citizens and chosen for this study are shown in Table 1.

**Table 1.** Local names and main ingredients of selected traditional dishes and Arabic sweets frequently consumed in Lebanon.

Traditional dish/ Arabic sweet	Ingredients
Baba ghanouj	Aubergines, garlic cloves, lemon juice, tahini, pomegranate seeds, salt
Batata mehchi	Lamb ground, onions, butter, salt, pepper, pine nuts, potato, tomato juice
Borgul bi banadoura	Coarse bulgur wheat, small pearl onions, chickpeas, cinnamon stick, caraway seed, vegetable oil, mild white pepper, salt
Chichbarak	Chichbarak Dough: multi-purpose flour, salt, water warm to form a paste, yeast, sugar Meat Stuffing: ground beef, salt to taste, black pepper to taste, cinnamon powder to taste, onion finely chopped, pine nuts, olive oil, bushel of parsley chopped Chich Barak Stew: yogurt, water, starch, garlic cloves crushed (optional), rice, dried mint, salt to taste
Falafel	Dry peeled fava beans dried chickpeas (aka Garbanzo beans), Italian parsley (chop away the stems), green cilantro (chop away the stems), freshly peeled crushed garlic cloves, red or yellow onion, green onions, salt, black pepper, flour, baking soda, red chili pepper (optional, if spicy falafel is desired), cumin, coriander Falafel Tahini Sauce: Tahini paste, freshly squeezed lemon juice, garlic cloves, crushed, salt
Fatayer sabanikh	Fresh spinach, onions, pine nuts, lemon juice, olive oil, sumac, salt, plain white flour, caster sugar, baker yeast, olive oil, salt
Fattat Hommos	Chickpeas, tomatoes, onion, basil leaves, garlic cloves, pitta bread, pine nuts, yogurt, tahini and vinegar, vegetable oil, salt and pepper
Fattoush	Lettuces or romaine lettuce, cherry tomatoes, cucumbers, radishes, spring onions, flat-leaf parsley mint, pitta bread, olive oil, vinegar, sumac, salt
Foul moudamas	Broad beans, baking soda, water, water, salt, garlic cloves minced, lemon juice, olive oil
Hindbe bi zet	Chicory greens, water, olive oil, onions, salt, lemon juice
Hommos bi tahini	Chickpeas, garlic cloves, lemon juice, Tahini, olive oil, salt
Kafta wa batata	Minced lamb, flat-leaf parsley, onions, salt and pepper, onions, red pepper, tomato juice, debs roman, olive oil, salt and pepper, potatoes, ripe tomatoes, vegetable oil
Kibba bi sayniya	Finely ground beef (or lamb, lean), bulgur cracked wheat, salt, all spice, cumin, onions (finely chopped)
Koussa mehchi	Minced lamb, small zucchini, short grain rice, olive oil, salt and black pepper
Lahm bi ajeen	Plain white flour, caster sugar, baker′s yeast, salt, olive oil, minced lamb, tomatoes, few drops of pomegranate molasses, salt and pepper
Loubia bi zet	Vegetable oil, white onions, sliced, frozen bag green beans, garlic cloves, peeled, Cans of chopped tomatoes, salt and sweet pepper to taste, 7 spices, extra-virgin olive oil
Malfouf mehchi	Cabbage leaves, basic vegetables stuffing, tomato, lemon juice, water, cinnamon, garlic cloves, dry mint
Moujadara	Green or coral lentils, short-grain rice, onions, olive oil, salt
Moghrabia	Dry dough, chickpeas, pearl onions, vegetable oil, caraway butter, ground cinnamon, ground cumin, salt, black pepper
Mousaka batinjan	Aubergine, yellow or white onion, diced, garlic cloves, minced, low-salt chickpeas, extra virgin olive oil, low-salt diced tomatoes, tomato paste, piquant post spicy mint blend, pita chips or crusty bread for dipping, salt and pepper to taste
Riz a dajaj	Chicken breast, basmati rice, carrot, onions, tomato juice, whole black peppercorns, whole green cardamoms, cinnamon, cloves, cumin, vegetable oil, salt
Riz b lahma	Medium fat meat, basmati rice, carrot, onions, tomato juice, whole black peppercorns, whole green cardamoms, cinnamon, cloves, cumin, vegetable oils, salt
Sayadiah	Sea bass, scaled and gutted or in fillets, basmati rice, onions, caraway seeds, ground cumin, pines nuts, olive oil, fish stock, vegetable oil, salt, flour, butter, lemon juice
Shawarma dajaj	Chicken, olive oil, onions, red vinegar, lemon juice, pepper, cinnamon, nutmeg, salt, cloves of garlic
Shawarma lahma	Meat, olive oil, onions, red vinegar, lemon juice, pepper, cinnamon, nutmeg, salt, cloves of garlic
Tabboula	Tomatoes, spring onions, flat leaf parsley, mint, bulgur wheat, lemon juice, olive oil, salt
Warak enab	Vine leaves, tomatoes, onion, flat-leaf parsley, mint, lemon juice, short-grain rice, meat, olive oil, salt
Yakhnet Bamia	Lamb cubed, onions, garlic cloves minced, green coriander, okra, lemon juice, salt, pepper, water and tomatoes
Yakhnet Fassoulia	Shoulder of lamb, fresh white haricot beans, coriander, onions, garlic cloves, tomato juice, olive oils, water or chicken stock, salt and black pepper
Yakhnet Mouloukhia	Free-range chicken, basmati rice, coriander, garlic cloves, onion, shallots, pitta bread, vinegar, lemon juice, vegetable oil and salt
Baklava mixed	Sheets of phyllo pastry, unsalted melted butter, fragrant sugar syrup. Filling Ingredients: hulled unsalted pistachios, superfine sugar, orange blossom water, rose water
Baklava mixed light	Sheets of phyllo pastry, unsalted melted butter, sugar alcohol. Filling Ingredients: hulled unsalted pistachios, sugar alcohol, orange blossom water, rose water
Barazik	Sesame seeds (lightly toasted), clarified chilled butter, icing sugar, egg, vanilla, vinegar, flour, baking powder, a pinch of salt, thinly sliced pistachios, milk
Boundoukia	Hazelnut, sugar, water, corn flour, salt, orange blossom water, rose water, butter
Daoukia	hulled unsalted pistachios, semolina, sugar, milk, orange blossom water, rose water, butter, green colorant
Foustoukia	Egg white, fine sugar, fine powdered sugar, blossom water, almonds, pistachio, dried flowers for decoration
Ghourayba	Organic sugar cane, small grains mastic to yield powdered mastic, egg yolks, unsalted butter at room temperature, unbleached all-purpose flour, blanched whole almonds.
Halawa	Unbleached all-purpose flour, grounded aniseed, grounded cinnamon, small grains mastic finely grounded to yield powdered mastic, sesame seeds, blanched toasted almonds, confectioners′ sugar, honey, unsalted butter, sunflower oil
Halawa light	Unbleached all-purpose flour, grounded aniseed, grounded cinnamon, small grains mastic finely grounded to yield powdered mastic, sesame seeds, blanched toasted almonds, Sugar alcohol, unsalted butter, sunflower oil
Halawet el jiben	Akkawi cheese, sugar, semolina, water, kashta, orange blossom water, rose water, sugar syrup, lemon blossom and grated pistachio for decoration
Ish el bulbul	Knefeh dough, melted butter, honey, kashta, sweetened lemon blossom, pistachios
Kallaj kashta	Sugar, pistachios, lemon blossom, milk, kallaj sheets. Kashta ingredients: milk, cream fresh, rose water, blossom water, corn flour, sugar. Sugar syrup ingredients: sugar, water, lemon juice
Karabij joz maa crema	Grounded finely walnuts, caster sugar, cold water, rose water, extra-fine semolina, unsalted softened butter, granulated sugar, grounded mahlab, whole milk, active dry yeast dissolved with sugar in water. Cream Ingredients: egg whites, sugar, rose water, blossom water
Katayef kashta	Flour, a pinch of salt, sugar, instant dry yeast, lukewarm water, baking soda, rose syrup. Akkawi cheese or ricotta mixed with Mozarella, fresh grated Mozarella cheese, sugar, rose water
Kounafa kashta bil kaak	Milk, cream fresh, rose water, blossom water, corn flour, sugar, ″hair″ pastry, unsalted diced butter, fragrant sugar syrup
Kounafa bil jiben	Akkawi cheese or cow′s milk mozarella, ″hair″ pastry, unsalted diced butter, fragrant sugar syrup
Maakaron	Blanched almonds plus whole almonds, superfine sugar, almond extract, freshly squeezed lemon juice, egg whites
Maakroun and moushabak	Flour, corn flour, yeast, water. Sugar syrup: sugar, water, lemon juice
Maamoul tamer	Semolina, grounded mahlab, clarified melted butter, instant dry yeast, sugar, full-fat milk, icing sugar for dusting. Chopped pitted dates, grounded nutmeg, melted butter
Maamoul mad kashta	Semolina, ghee, sugar, fine semolina, ground mahlab, yeast, rose water, blossom water. Filling ingredients: water, sweetened condensed milk, corn flour, blossom water
Maamoul mad joz	Semolina, flour (ferkha), unsalted butter melted, ghee, melted, instant yeast, flour, sugar, orange blossom water, rose water For filling: walnuts, pistachios, orange blossom water, rose water, sugar
Maamoul fostok	Regular semolina, unbleached all-purpose flour, superfine sugar, fast-acting (instant) yeast, unsalted butter at room temperature, orange blossom water, rose water. Hulled unsalted pistachio, superfine sugar, orange blossom water, rose water
Maamoul joz	Semolina, grounded mahlab, clarified melted butter, instant dry yeast, sugar, full-fat milk, icing sugar for dusting Fine chopped walnuts, sugar, orange blossom water, zest of orange, and cinnamon
Madlouka	Milk, starch, sugar, semolina, fried nuts almond and cashew, vinegar, blossom water, ghee, water, ground pistachio liquid cream, lemon juice, sugar cherry
Mafrouka kashta	Semolina, cream liquid milk, red lemon blossom, ground pistachio, flower water, liquid whipping cream, white toast, butter, liquid vanilla, corn starch Sugar syrup ingredients: sugar, water
Mafrouka fostok	Sugar, butter, sweetened concentrated milk, rose water, water, semolina, roasted pistachio mixture, and cornstarch. Sugar syrup ingredients: sugar, water, rose water
Moufattaka	Rice, sugar, tahini, turmeric, water, pine nuts, cinnamon powder
Mouhallabiya	Whole milk, granulated sugar, liquid honey (optional), grounded almonds, grounded walnuts
Moushabak	Flour, corn stretch, water, dry instant yeast, sugar, sunflower oil for frying. Sugar syrup
Nammoura	Regular semolina, fine semolina, grounded mastic with sugar, sugar, salt, clarified melted butter, rose water, yogurt, baking soda, tahini, blanched almonds, rose syrup. Sugar, water, lemon juice, orange blossom water, rose water
Osmaliya	Knefeh packet, milk, starch, melted ghee, sugar syrup, crushed pistachios
Riz bil Halib	Cornstarch, cold milk, full-fat milk, heavy cream, vanilla pod, presoaked and drained short-grain white rice, sugar, rose water, orange blossom water
Saniora	Flour, sugar, ghee, butter, almonds or pistachios
Sfouf	Semolina, flour, turmeric powder, milk, vegetable oil, sugar, water, baking powder, tahina to grease the baking tray, pine nuts
Shaaybiyat	Sheets of phyllo pastry, unsalted melted butter, fragrant sugar syrup. Walnuts, superfine sugar, grounded cinnamon, orange blossom water, rose water. Superfine sugar, freshly squeezed lemon juice, water, rose water, orange blossom water
Ward el sham	Phyllo pastry sheets, cream, butter, vegetable oil, ghee, ground pistachios. Sugar syrup ingredients: sugar, water, lemon juice, rose water, blossom water
Znoud el sitt	Spring roll dough, milk, liquid cream, starch, flour, water, rose water, corn oil, sugar syrup, pistachios

### Data collection

A sample of 500 g of each dish or sweet was collected and used for analysis. According to Greenfield *et al.*, this size is a convenient sample to avoid errors during analysis ^15^. Our research group collected 500 g of 30 types of traditional dishes from a central kitchen in Beirut and 35 types of Arabic sweets from popular sweet retails in the same area. These popular kitchen and sweet retailers, which serve traditional meals and Arabic sweets were found on the internet and chosen based on the following criteria: 1) their specialty in cooking home-made dishes and serving Arabic sweets; 2) their popularity; and 3) the popular kitchen’s involvement in supporting women, as part of social entrepreneurship initiatives that are aimed at empowering women. Regarding traditional meals, five samples for each dish were collected previously from various regions of Lebanon and tested to eliminate any discrepancies ^16^. The laboratory analyses of the 150 samples showed, after being tested using Chi-square, no significant differences in all of the variables tested (p=0.4, data not shown) between different governorates in Lebanon. IBM SPSS Statistics 26.0 was the software used for analysis of the results.

### Chemical analysis

After the receipt of the food samples, 500 g of each composite dish was mashed and then analyzed in the laboratory. The remaining samples were kept frozen at -18°C in tight containers for further analysis. According to the standard Association of Official Agricultural Chemists ^17^ procedures, components of the dishes such as ash, moisture, crude protein and crude fat were analyzed. Using thermal drying methods with a Fisher Isotemp vacuum oven, moisture was identified. The food samples were heated to 105°C and the measurement of moisture content was based on the loss of weight of the sample. According to the International Dairy Federation Standard, crude protein was calculated through the multiplication of 6.38 by total Kjeldahl nitrogen (AOAC 991.20-23) ^17^. Total fatty acid was analyzed by extraction with petroleum ether using the Soxhlet extraction system. The Roese-Gotlieb method was used in the investigation of the fat content (AOAC 945.48, 933.05 & 963.15, 2019) ^17^. To obtain ash, using an Isotemp muffle furnace, an oxidation of all organic matter in a weighed sample was achieved by incineration in a muffle furnace at 550°C overnight; then the weight of the remaining ash was measured. CHO was calculated by subtracting the sum of the percentages of the measured weights of fat, protein, moisture, and ash from the total weight (100g). Energy was expressed in kilocalories (kcal). Using Atwater calorie conversion factors, calorie values were calculated based on the total grams of protein, fat, and CHO, as 4, 4, and 9 kcal/g respectively ^18^.

### Development of exchange lists

The macronutrient exchanges were determined based on the laboratory values provided from the analysis of 100 g of each dish. Wheeler and collaborators (Wheeler *et al.* 1996) ^19^ described a round-off method which was used to yield exchange numbers. The macronutrient exchanges were calculated as follows:

***CHO exchange.*** The dish was not considered as one serving if it contained 1–5 g carbohydrate. If the food portion contained 6–10 g CHO, the dish was considered as half a serving and if it contained 11–20 g CHO, it was considered as one serving.

***Protein exchange.*** If the amount of protein ranged between 0–3 g in the meat and meat substitute dishes, it was not counted as a serving. If it contained 4–10 g protein, it was considered as one serving.

***Fat exchange.*** If the amount of fat in food portions ranged between 0–2 g, it was not considered as a serving. However, if the dish contained 3g of fat, it was counted as half a serving and if it contained between 4 and 7 g fat, it was counted as one serving. Moreover, the amount of the meal (in grams) that yielded one CHO, one protein and one fat exchange was obtained by the calculation of CHO grams, protein grams, and fat grams by 15, 7, and 5, respectively.

## Results and discussion

The results of the analysis of 100g from each dish and Arabic sweet are presented in Table 2.

**Table 2.** Composition of 100 g of traditional dishes and Arabic sweets in terms of carbohydrate (CHO), fat, protein, ash, moisture and energy and the percentage contribution to a 2000 Kcal diet of 100 g of traditional dishes and Arabic sweets for the amount of the same variables. The daily recommended amount is 275 g/d for CHO (55%), 50g/d for protein (10%) and 78g/d for fat (35%).

	Amounts in 100 g of Edible Portion (per gram)						% DV in 2000 Kcal (100 g)			
Traditional Dish/ Arabic sweets	Moisture	Ash	CHO	Protein	Fat	Energy	CHO	Protein	Fat	Energy
Baba ghanouj	91.5	1.1	4.5	1.1	1.8	39	1.6	2.2	2.3	1.9
Batata mehchi	69.5	1.8	18	5	5.7	143	6.5	10	7.3	7.1
Borgul bi banadoura	69.1	1.5	20.8	3	5.6	146	7.5	6	7.1	7.3
Chichbarak	68.1	1.7	18.7	4.8	6.7	154	6.8	9.6	8.5	7.7
Falafel	31.3	3.4	36.5	13.3	15.6	339	13.2	26.6	20	16.9
Fatayer sbanikh	45	2.4	27.2	5.3	20.1	311	9.8	10.6	25.7	15.5
Fattat hommos	68.7	1.3	15.8	6.5	7.7	159	5.7	13	9.8	7.9
Fattoush	88.1	1.3	7.2	1.5	1.9	52	2.6	3	2.4	2.6
Foul moudamas	75.3	1	14.2	5.3	4.2	116	5.1	10.6	5.3	5.8
Hindbe bi zet	67.7	1.5	5.9	2.5	22.4	235	2.1	5	28.7	11.7
Hommos bi tahini	68.2	1.9	17.2	7.5	5.2	146	6.2	15	6.6	7.3
Kafta wa batata	79.3	1.5	7	8.8	3.4	94	2.5	17.6	4.3	4.7
Kibba bi sayniya	51.4	1.8	19.7	11.3	15.8	266	7.1	22.6	20.2	13.3
Koussa mehchi	71.6	1.4	20.3	3.8	2.9	123	7.3	7.6	3.7	6.1
Lahm bi ajeen	44.6	1.5	37.1	11.2	5.6	244	13.4	22.4	7.1	12.2
Loubia bi zet	86.5	1.4	7.2	2.1	2.8	62	2.6	4.2	3.5	3.1
Malfouf mehchi	81.5	1.3	12.1	3.8	1.3	75	4.4	7.6	1.6	3.7
Moujadara	71.1	1.2	21.8	5.4	0.5	113	7.9	10.8	0.6	5.6
Moghrabia	72.8	1	15.6	6.7	3.9	124	5.6	13.4	5	6.2
Mousaka batinjen	70.6	1.1	14.8	3.2	10.3	165	5.3	6.4	13.2	8.2
Riz a dajaj	65.4	1.6	18.8	7.2	7	167	6.8	14.4	8.9	8.3
Riz bi lahma	63	1.7	23	7.5	4.8	165	8.3	15	6.1	8.2
Sayadiah	64.2	0.9	22.1	6.5	6.3	171	8	13	8	8.5
Shawarma dajaj	58.6	2.4	1.1	29.7	8.2	197	0.4	59.4	10.5	9.8
Shawarma lahma	67.3	1.6	2.6	17.5	11	179	0.9	35	14.1	8.9
Tabboula	88.3	1.4	6.1	1.9	2.3	53	2.2	3.8	2.9	2.6
Warak enab	75.1	1.3	17.7	4.4	1.5	102	6.4	8.8	1.9	5.1
Yakhnet Bamia	73.5	1.3	17	3.9	4.3	122	6.1	7.8	5.5	6.1
Yakhnet Fassoulia	66.3	1.1	22.6	8.1	1.9	140	8.2	16.2	2.4	7
Yakhnet Mouloukhia	76.3	1.6	11.9	5.4	4.8	112	4.3	10.8	6.1	5.6
Baklava mixed	7	1.1	64	6.6	27.3	474	23.2	13.2	28	23.7
Baklava mixed light	7.6	1	61.9	7.1	28.7	478	22.5	14.2	29.4	23.9
Barazik	1.5	1.3	49	15.3	42.1	553	17.8	30.6	43.2	27.7
Boundoukia	4.8	1.5	64.1	11.5	23.2	465	23.3	23	23.8	23.3
Daoukia	27.4	0.7	52.8	7.3	15.1	347	19.2	14.6	15.5	17.4
Foustoukia	4.8	1.8	59.7	19.2	18.5	446	21.7	38.4	19	22.3
Ghourayba	4.2	0.4	62.9	6.8	32.9	510	22.8	13.6	33.8	25.5
Halawa	3.8	1.7	45.7	16.8	41	538	16.6	33.6	42.1	26.9
Halawa light	0.7	1.4	57.5	12.6	35.6	531	20.9	25.2	36.5	26.6
Halawet El Jiben	45.4	1.3	36.7	9.7	8.8	248	13.3	19.4	9	12.4
Ish el bulbul	5.2	1	65.8	7.5	26.2	478	23.9	15	26.9	23.9
kallaj kashta	55	0.8	33	3.6	9.7	215	12	7.2	10	10.8
karabij joz maa crema	17.8	0.6	61.1	7.6	16.5	391	22.2	15.2	16.9	19.6
katayef kashta	44.5	0.8	40.5	6	10.5	260	14.7	12	10.7	13
kounafa kashta bil kaak	42	0.9	42.2	8.1	8.7	262	15.3	16.2	8.9	13.1
Kounafa bil jiben	42.6	0.6	40.4	6.1	13.2	279	14.6	12.2	13.5	14
Maakroun and moushabbak	5.2	0.2	77.1	3.5	17.9	448	28	7	18.4	22.4
Maamoul tamer	11.8	1	68.4	6.6	15.6	410	24.8	13.2	16	20.5
Maamoul mad kashta	37.2	0.9	49.6	5.3	8.9	283	18	10.6	9.2	14.2
Maamoul mad joz	12.9	0.8	58.8	8	25	443	21.3	16	25.6	22.2
Maamoul fostok	14.5	0.8	53.3	10.4	26.9	444	19.3	20.8	27.6	22.2
Maamoul joz	8.9	0.6	66.2	10	18.3	433	24	20	18.8	21.7
Madlouka	29.36	1.1	51.1	8	13	328	18.5	16	13.4	16.4
Mafrouka kashta	10.1	0.8	71.1	4.3	17.5	425	25.8	8.6	18	21.3
Moufattaka	26.3	0.7	59.2	5.9	10.1	332	21.5	11.8	10.3	16.6
Mouhallabiya	55.4	1.1	31.6	6.2	7.3	203	11.4	12.4	7.5	10.2
Moushabak	13.5	<0.1	71.5	2.1	16.4	410	26	4.2	16.8	20.5
Nammoura	14.3	0.4	75.4	3	8.8	376	27.4	6	9	18.8
Osmaliya	48.4	1.3	27	9.3	17.9	271	9.8	18.6	18.4	13.6
Riz bil halib	54.2	1.3	32.7	6.7	6.5	204	11.8	13.4	6.7	10.2
Saniora	2.3	1	68.7	7.4	26.4	490	24.9	14.8	27.1	24.5
Sfouf	20.6	0.7	55.2	6.3	22	401	20	12.6	22.6	20.1
Shaaybiyat	39.4	0.5	39.1	9.5	14.7	298	14.2	19	15.1	14.9
Ward el sham	46.9	1.2	34.4	8.2	11.9	254	12.5	16.4	12.2	12.7
Znoud el sitt	40.3	0.3	41	4.5	17.8	307	14.9	9	18.2	15.4

### Traditional dishes

The amount of moisture ranged from 31.3 % in *Falafel* to 91.5% in *Baba Ghanouj*. The protein content was lowest in *Baba Ghanouj* (1.1 %) and highest in *Shawarma Dajaj* (29.7%). *Hindbeh bi zeit*, which is fried chicory with onions, contained the top increased fat content (22.4%) of the analyzed dishes and was greater than the 6.7 % shown previously in 1970 in Lebanon ^11^. *Falafel*, a fried dish, has the highest energy value (339 kcal/100 g). On the other hand, almost all the stews such as *Yakhnet Bamia*, *Yakhnet Fassoulia* and *Yakhnet Mouloukhia* have medium levels of energy ranging between 110 and 140 kcal per 100 g ( Table 2). On the other hand, although the amount of energy in the dishes was identical, the dissimilarity in protein, CHO or fatty acid content had nutritional implications on health, since a high intake of CHO or fats is associated with a high-risk factor for non-communicable diseases ^5^.

The nutrient goal represents the average intake that is compatible with the maintenance of good health in individuals ^20^. According to the US Food and Drug Administration (FDA) definition, the daily value (DV) is described as the “reference values for reporting nutrients on the nutrition labels”. The percentage (%) of DV assists the consumer in recognizing how the serving of food, and its content in nutrients, fits into their daily diet. As per FDA regulations, the expression “high,” “rich in,” or “excellent source of” nutrients are used if the food has ≥20% of the daily value per reference amount. The terms “good source,” “contains,” or “provides” are used if the food yields 5–19% of the recommended dietary intake (RDI) per reference amount of the nutrient. Foods that carry <5% of the RDI from the nutrient per reference amount are considered to have low amounts. In our study, the contribution of each dish (per 100g) to the overall amount of CHO, protein and fat needed per day was calculated. The calculations are presented in Table 2.

Pellet *et al.*, in 1970, showed high total fatty acid content in *Lahm bi ajin* (39.4%) which was higher than the reported value of 8% in our study ^11^. There is limited available research on the composition of Lebanese traditional composite dishes, thus the results provided in this study were compared with data from other countries in the EMR ^11, 21– 26^, mainly the amount of total fatty acid content in these foods. The amount of total fatty acid in the foods consumed in Lebanon, Bahrain, Kuwait, Jordan and Saudi Arabia are extremely important given the elevated prevalence of non-communicable diseases in the countries ( Table 3) ^26^. Compared to our findings, increased fatty acid content was observed in *Falafel* that was also reported in many other Arab countries (14.3% in Saudi Arabia to 18.4% in Jordan) ^23, 24^. The fatty acid content in *Baba Ghanouj* was double the level described in Jordanian *Baba Ghanouj* (9.4% and 5.4%, respectively) ^23^ and triple the level reported previously in 1970 in the Lebanese *Baba Ghanouj*. Furthermore, the Kuwaiti *Baba Ghanouj’s* fatty acid content was moderately lower than that described in our study (8.7 and 9.4, respectively) ^22^. Total fatty acid levels in *Batata Mehchi* ranged from 5.6% in Lebanon at 1970 to 5.9 % in Saudi Arabia ^11, 24^. Musiager *et al.*, in 1998 ^21^, found double the amount of fatty acid levels in *Bourgul bi banadoura*, *Chichbarak and yakhnet Bamia when* compared to our study ( Table 3). The high fatty acid content of *Fatayer Sabanikh* was shown in almost all other studies enrolled in Arab countries; the content found in our findings was higher than all values reported in all countries ( Table 3). In our study, *Fattoush* contained lower fatty acid content compared to the same dish of Kuwait origin (2.94% and 2.17%, respectively) ^22^. In contrast, our results contradicted the values reported previously in Lebanon and in Jordan (6.3% and 8.6% respectively) ^11, 23^. Since *Foul Moudamas* is frequently consumed with added olive oil in Lebanon, thus, the availability of total fatty acids is high in this dish. The findings of our study show that the amount of fatty acid in *Foul Moudamas* was similar to those prepared in all the Arab countries except for Jordan ^23^. The values of fat in the remaining dishes are shown in Table 2. Since the protein and CHO content of the meals studied were not explored in all the Arab country-based studies, it is impossible to compare these variables to our findings.

**Table 3.** List of fat composition of food commonly tested in Lebanon, Kuwait, Jordan, Saudi Arabia and Bahrain. NA: Non available; *Pellet, 1970; ^^^Musaiger, 1998; ^@^Dashti, 2001; ^Ω^ Bawadi, 2009; ^€^Alfares, 2018; ^¥^Musaiger, 2011.

Traditional Dish/ Arabic sweet	Lebanon 1970 *	Lebanon 1998 ^^^	Lebanon 2020	Kuwait 2001 ^@^	Jordan 2009 ^Ω^	Saudi Arabia 2018 ^€^	Bahrain 2011 ^¥^
	Fat in 100 g	Fat in 100 g	Fat in 100 g	Fat in 100 g	Fat in 100 g	Fat in 100 g	Fat in 100 g
Baba ghanouj	3.7	NA	9.44	8.7	5.4	NA	NA
Batata mehshi	5.6	NA	1.24	NA	NA	5.9	NA
Bourghol b banadoura	NA	10.16	5.02	NA	NA	NA	NA
Chichbarak	NA	8.89	4.6	NA	NA	NA	NA
Falafel	NA	NA	11.7	NA	18.4	14.3	NA
Fatayir sabanikh	6.6	NA	11	6.12	7.6	2.75	7
Fattoush	6.3	NA	2.94	2.17	8.6	NA	NA
Foul moudamas	3.1	NA	3.48	3.15	7.3	3.2	NA
Hindbe b′zeit	6.7	NA	10.7	NA	NA	NA	NA
Hommus bi tahini	19.7	NA	6.4	7.7	NA	17.8	NA
Mosakaa batinjen	NA	NA	6.58	NA	NA	16.4	NA
Kafta	22.1	NA	6.32	NA	NA	NA	NA
Kussa mehshi	1.7	NA	2.42	NA	NA	NA	NA
Lahm b ajin	39.5	NA	8	NA	NA	NA	NA
Malfuf mehshi	2.6	NA	2.12	NA	NA	NA	NA
Mjaddara	5.6	NA	5.8	NA	NA	NA	NA
Riz a djeij	9.3	NA	5.4	NA	NA	NA	NA
Sayadiyah	13.2	NA	6.48	3.98 (sandwich)	NA	NA	NA
Shawarma djeij	NA	NA	6.94	3.90 (sandwich)	NA	NA	11.2
Shawarma lahma	36	NA	8.28	NA	14	NA	9.4
Tabboula	5.8	NA	4.24	3.3	2.6	NA	NA
Warak enab	7.3	NA	3.98	NA	NA	3.7	NA
Yakhnet bamiah	7.2	11.09	5.4	NA	NA	NA	NA
Yakhnet fassoulia	6.6	NA	3.9	NA	NA	NA	NA
Yakhnet mloukhia	6.4	NA	4.28	NA	NA	0.25	NA
Baklava mixed	NA	NA	27.3	NA	28.7	NA	38.8
Barazik	NA	NA	42.1	NA	34.6	NA	NA
Ghouraybah	21.3	NA	32.9	NA	28	NA	NA
kallaj kashta	NA	NA	9.7	NA	25.8	NA	NA
katayef kashta	NA	NA	10.5	NA	7.1	NA	NA
Knefah b jibn	NA	NA	13.2	NA	21.6	NA	NA
Maakroun and mshabbak	17.2	NA	17.9	NA	NA	NA	NA
Maamoul tamer	9	NA	15.6	NA	16.3	10	NA
Maamoul fostok	NA	NA	26.9	NA	19.3	NA	NA
Maamoul joz	NA	NA	18.3	NA	27.3	NA	NA
Nammoura	3.1	NA	8.8	NA	18.4	NA	NA

The macronutrient exchanges yielded from the analysis of 100 g of each of the 30 dishes are shown in Table 4. In addition, the serving size of each dish which would provide one exchange of each macronutrient was calculated ( Table 4). At least one exchange of starch was found in almost all dishes except *Baba Ghanouj, Fattat homos, Fattoush, Hindbe bil zet, Loubia bil zet, Shawarma Dajaj, Shawarma Lahma, Yakhnet Mouloukhiya and Tabboula.* The bean’s stew ( *Yakhnet Fassoulia*) contained the highest amount of CHO exchanges (1.5 exchanges). The highest numbers of fat exchanges are found in *Mousaka Batinjan*, *Fatayer sabanikh* and *Hindbe bil zet* ( Table 4). Less than 10 g of protein per portion size was determined in all dishes except for *Kafta wa Batata* and *Shawarma Dajaj* ( Table 4).

**Table 4.** Exchange list and serving sizes of Lebanese traditional dishes and Arabic sweets. WM: whole milk; MFM: medium fat meat; LM: lean meat; HFM: high fat meat; RFM: reduced fat meat; Tbsp: Table spoon; 1 Tbsp: 15 g.

	Serving Exchanges per 100g	Serving size per measurement tool	Exchange per serving size	Amounts per Serving in gram and Kcal			
Appetizer/Dish				CHO	Protein	Fat	Energy
**Baba ghanouj**	1 vegetable, 0.25 fat	100 g (6 Tbsp)	1 vegetable, 0.25 fat	4.5	1.1	1.8	39
**Batata mehchi**	1,25 starch, 1 fat	100 g (1 Large or 3 small)	1,25 starch, 1 fat	18	5	5.7	143
**Borgol bi banadoura**	1,5 starch, 0.5 fat	100 g (1/2 cup)	1,5 starch, 0.5 fat	20.8	3	5.6	146
**Chichbarak**	1 starch, 0.5 WM	100 g (1/2 cup)	1 starch, 0.5 WM	18.7	4.8	6.7	154
**Falafel**	2 starch, 2 MFM, 0.5 fat	40 g (2 patty balls)	1 starch, 1 MFM	14.6	5.3	6.2	135.6
**Fatayer sbanikh**	1.5 starch, 1 protein, 4 fat	55 g (1 triangle)	1 starch, 2 fat	14.9	2.9	11.0	171.0
**Fattat hommos**	1 whole milk	100 g (1/2 cup)	1 WM	15.8	6.5	7.7	159
**Fattoush**	1 vegetable, 0.5 fat	200 g (1 cup)	2 vegetable, 1 fat	14.4	3	3.8	104
**Foul moudamas**	1 starch, 1 LM	100 g (1/2 cup)	1 starch, 1 LM	14.2	5.3	4.2	116
**Hindbe bil zet**	1 vegetable, 4,5 fat	50 g (1/4 cup)	0.5 vegetable, 2 fat	2.9	1.2	11.2	117.5
**Hommos bi tahini**	1 starch, 1 MFM	100 g (6 Tbsp)	1 starch, 1 MFM	17.2	7.5	5.2	146
**Kafta wa batata**	0.5 starch, 1 LM, 0.25 fat	200 g (1 cup)	1 starch, 2 LM, 0.25 fat	14	17.6	6.8	188
**Kibba bi sayniya**	1,25 starch, 1,5 HFM	76 g (half a square)	1 starch, 1 HFM, 0.5 fat	14.9	8.5	12.0	202.1
**Koussa mehchi**	1 starch, 1 vegetable, 0.5 fat	100 g (2 Medium)	1 starch, 1 vegetable, 0.5 fat	20.3	3.8	2.9	123
**Lahm bi ajeen**	2,5 starch, 1 LM	40 g (2 medium piece)	1 starch, 0.5 LM	14.8	4.4	2.24	97.6
**Loubi bil zet**	1,5 vegetable, 0.5 fat	100 g (1/2 cup)	1.5 vegetable, 0.5 fat	7.2	2.1	2.8	62
**Malfouf mehchi**	1 vegetable, 0.5 starch	100 g (4 pieces)	1 vegetable, 0.5 starch	12.1	3.8	1.3	75
**Moujadara**	1 starch, 0.5 LM	100 g (1/2 cup)	1 starch, 0.5 LM	21.8	5.4	0.5	113
**Moghrabia**	1 starch, 1 LM	100 g (1/2 cup)	1 starch, 1 LM	15.6	6.7	3.9	124
**Mousaka batinjan**	1 starch, 2 fat	100 g (1/2 cup)	1 starch, 2 fat	14.8	3.2	10.3	165
**Riz a dajaj**	1 starch, 1LM, 1 fat	100 g (1/2 cup)	1 starch, 1 LM, 1 fat	18.8	7.2	7	167
**Riz bi lahma**	1 starch, 1 LM, 1 fat	65 g (1/3 cup)	1 starch, 0.5 LM	14.9	4.8	3.1	107.2
**Sayadia**	1, 25 starch, 1 MFM	100 g (1/2 cup)	1, 25 starch, 1 MFM	22.1	6.5	6.3	171
**Shawarma dajaj**	4,25 LM	54 g (4 Tbsp)	2 LM,0.25 fat	0.5	16.0	4.4	106.3
**Shawarma lahma**	2,25 MF, 0.25 fat	50 g (4 Tbsp)	1 MF, 0.25 fat	1.3	8.7	5.5	89.5
**Tabboula**	1 vegetable, 0.5 fat	200 g (1 cup)	2 vegetable, 1 fat	12.2	3.8	4.6	106
**Warak enab**	1 vegetable,1 starch	100 g (6 pieces)	1 vegetable,1 starch	17.7	4.4	1.5	102
**Yakhnet bamia**	0.5 starch, 2 vegetable, 1 fat	100 g (1/2 cup)	0.5 starch, 2 vegetable, 1 fat	17	3.9	4.3	122
**Yakhnet fassoulia**	1,5 starch, 1 LM	100 g (1/2 cup)	1,5 starch, 1 LM	22.6	8.1	1.9	140
**Yakhnet mouloukhia**	2 vegetable, 0.5 LM, 1 fat	100 g (1/2 cup)	2 vegetable, 0.5 LM, 1 fat	11.9	5.4	4.8	112
**Baklava mixed**	3 starch, 1 sugar, 4 fat	23 (1 piece)	1 starch, 1 fat	14.7	1.51	4.8	109
**Baklava mixed light**	3,25 starch, 4 fat, 0.5 sugar	24 (1 piece)	1 starch, 1 fat	14.8	1.7	5.3	114
**Barazik**	3 starch, 1 LM, 6 fat	30 (1 piece)	1 starch, 2 fat	14.7	4.5	9.8	165
**Boundoukia**	3 starch,1 LM, 3 fat, 1 sugar	23 (1 medium bar)	1 starch, 1 fat	14.7	2.6	4.1	106
**Daoukia**	2.5 starch, 1 RFM, 1 fat	28 (1 small piece)	1 starch, 0.5 fat	14.7	2	3.3	97
**Foustoukia**	4 starch, 1 LM, 2 fat	25 (1 medium bar)	1 starch, 1 fat	14.9	4.8	3.6	111
**Ghourayba**	3 starch, 1 sugar, 5 fat	24 (2 pieces)	1 starch, 1 fat	15	1.6	6.1	117
**Halawa**	3 starch, 1 LM, 6 fat	33 (2 Tbsp)	1 starch, 2 fat	15	5.5	10.5	172
**Halawa light**	4 starch, 5 fat	26 (1.5 Tbsp)	1 starch, 1,5 fat	14.9	3.2	7.2	138
**Halawet el Jiben**	1 starch, 1 RFM, 1 sugar	41 (2 medium pieces or 1 large piece)	1 starch, 0.25RFM	15	3.9	2.8	99
**Ish el bulbul**	3 starch, 1 sugar, 4 fat	23 (1 piece)	1 starch, 0.5 fat	15.1	1.7	4.7	105
**kallaj kashta**	2 starch, 1.5 fat	45 (1 large piece)	1 starch, 0.5 fat	14.8	1.6	3.4	96
**karabij joz maa** **crema**	4 starch, 1 LM, 1 fat	24 (2 pieces)	1 starch, 0.5 fat	14.6	1.8	3	93
**katayef Kashta**	2 starch, 1 HFM	37 (1 small piece)	1 starch, 0.5 fat	14.9	2.2	3	96
**kounafa kashta bil** **kaak**	1.75 starch, 1 RFM	35 (1 small piece or 2 Tbsp)	1 starch, 0.25 fat	14.7	2.8	2.3	91
**Kounafa bil jibn**	2 starch, 1 MFM, 1 fat	37 (1 small piece or 2 Tbsp)	1 starch, 0.5 fat	14.9	2.2	3.8	103
**Maakroun and** **moushabbak**	3 starch, 2 fat, 2 sugar	19 (1 small piece from each one)	1 starch	14.6	0.6	2.6	85
**Maamoul tamer**	3 starch, 2 fat, 1 sugar	22 (2 small pieces)	1 starch, 0.5 fat	15	1.4	2.6	90
**Maamoul mad** **kashta**	3 starch, 1 fat	30 (1 small piece)	1 starch	14.8	1.5	2.1	85
**Maamoul mad joz**	2,5 starch, 1 sugar, 4 fat	25 (1 small piece)	1 starch, 1 fat	14.7	2	4.8	110
**Maamoul fostok**	3,5 starch, 4 fat	28 (1 small piece)	1 starch, 1 fat	14.9	2.9	5.8	124
**Maamoul joz**	3 starch, 2.5 fat, 1 sugar	22 (1 small piece)	1 starch, 0.5 fat	14.5	2.2	3.1	95
**Madlouka**	2.25 starch, 1 WM	29 (2 Tbsp)	1 starch, 0.5 fat	14.8	2.3	2.9	95
**Mafrouka kashta**	3,5 starch, 1 sugar, 2 fat	21 (1.5 Tbsp)	1 starch	14.9	0.9	2.8	89
**Moufattaka**	2 starch, 2 sugar, 1 fat	25 (1.5 Tbsp)	1 starch	14.8	1.4	1.9	83
**Mouhallabiya**	1 other CHO, 1 RFM, 0.5 sugar	47 (3Tbsp)	1 other-CHO, 0.25 RFM	14.8	2.9	2.6	95
**Moushabak**	2.5 starch, 2 fat, 2 sugar	21 (1 large piece)	1 starch	15	0.4	2.6	82
**Nammoura**	3 starch, 0.25 fat, 2 sugar	20 (1 small piece)	1 starch	15	0.6	1.3	75
**Osmaliya**	1 starch, 1 WM, 1 fat	55 (1 medium piece)	1 starch, 1 fat	14.8	5.1	7.7	149
**Riz bil Halib**	1 other-CHO, 1 RFM, 0.5 sugar	46 (3Tbsp)	1 other-CHO, 0.25 RFM	15	3	2.3	92
**Saniora**	3 starch, 1,25 sugar, 4 fat	22 (1 medium piece)	1 starch, 1 fat	15.1	1.6	4.5	107
**Sfouf**	3,25 starch, 3 fat	27 (1 small piece)	1 starch, 1 fat	14.9	1.7	4.6	108
**Shaaybiyat**	1.5 starch, 1 RFM, 1,5 fat	38 (1 small piece)	1 starch, 0.5 RFM	14.8	3.6	4.3	113
**Ward el sham**	1.25 starch, 1 WM	44 (1 medium piece)	1 starch, 0.5 fat	15.1	3.6	4	109
**Znoud el sitt**	2,5 starch, 2.5 fat	36 (1 medium piece)	1 starch, 1 fat	14.7	1.6	5	110

### Arabic sweets

The amount of moisture ranged between 0.7% in *Halawa light* to 55.4% in *Mohallabiya*. The highest amount of protein was observed in *Foustoukia* (19.2%) and the lowest amount was in *Moushabak* (2.1%). *Barazik*, which is a sesame cookie cooked with butter, contains predominantly more than 40% of fat and had the highest energetic content (553 kcal/100 g). on the other hand, the least energy-dense foods were puddings ( *Riz bi halib* and *Mohallabiya*).

As stated before, there is a gap in the research field on the composition of Lebanese traditional composite dishes and Arabic sweets, thus our findings were compared with data from other countries in the EMR ^11, 21– 26^, mainly the amount of total fatty acid content in these sweets. Compared to our findings, the fatty acid content of *Baklava* in Lebanon did not differ to that in Jordan; however, it was lowest than the value reported in Bahrain. Lebanese *Barazik, Ghourayba, Katayef Kashta, Maakaron and Moushabbak* and *Maamoul fostok* had the highest fatty acid content compared to other EM countries ( Table 3).

The macronutrient exchanges yielded from the analysis of 100 g of each of the 35 types of Arabic sweets are shown in Table 4. In addition, the serving size of each dish which would provide one exchange of each macronutrient was calculated ( Table 4). At least one exchange of starch was found in all Arabic sweets *. Barazik and Halawa* contained the highest amount of fat exchanges (2 exchanges per serving). In addition, less than 5 g of protein per portion size was determined in all Arabic sweets ( Table 4). These exchange lists for traditional dishes and Arabic sweets will assist healthcare professionals and dietitians in organizing culturally appropriate planning of healthy food, especially for those with non-communicable diseases. Furthermore, for effective medical nutritional therapy, these exchange lists may assist in monitoring food portions for these traditional dishes. In addition, the chemical composition of traditional foods is highly necessary in order to investigate the dietary consumption of populations ^25^ and explore the impact of healthy food consumption on disease prevention ^26^. 

The principal limitation of this study is that the dishes and Arabic sweets analyzed were commercially prepared and the dishes’ ingredients were not reported, only recipes. Table 1 provides the ingredient quantities available from Lebanese cookbooks; however, all the relevant findings in our study were analyzed in an accredited laboratory. In addition, there are differences in ingredient proportions and cooking methods among various countries in the Arabian Middle Eastern and Gulf regions ^22, 27^.

Despite these limitations, this study provides healthcare professionals and consumers with an updated food composition table and a new exchange list of Lebanese traditional dishes and Arabic sweets consumed in Eastern Mediterranean countries by providing the laboratory composition of 30 frequently consumed traditional foods and 35 frequently consumed Arabic sweets. This can help improving diet quality, the achieving weight loss and implementing self-control in obese or overweight individuals and/or individuals with diabetes.

## Conclusions

To conclude, the Lebanese food exchange lists for the 30 frequently consumed Middle Eastern traditional dishes and the 35 mostly consumed Arabic sweets are now available ^28^. This guide is a good source of information about the macronutrient content of traditional dishes and Arabic sweets cooked in Lebanon. It is important to consumers, dietitians, and researchers and it offers accessible, user-friendly, practical models and uses household measures that allow consumers, dietitians and healthcare professionals to develop meal plans with healthier selections. Jordan, Syria and Palestine also can get the maximum benefit from this work because of the similarity in their traditional dishes. Finally, Lebanese cuisine offers a wide variety of recipes rich in micronutrients which could prevent the rise in NCDs. Thus, data on micronutrients in traditional dishes and Arabic sweets would be of greater importance in halting the rise of diet-related NCDs in the EMR. Efforts like this will provide a solid framework for the implementation of nutrition policies and practices in the region.

## Data availability

OSF: Development of a Lebanese food exchange system based on frequently consumed Eastern Mediterranean traditional dishes and Arabic sweets

https://doi.org/10.17605/OSF.IO/QKFN8 ^28^

This project contains the following underlying data:

- Data-F1000Research-exchange list-traditional dishes.xlsx
- Excel-Arabic sweets Exchange-F1000research.xlsx

Data are available under the terms of the Creative Commons Zero “No rights reserved” data waiver (CC0 1.0 Public domain dedication).
