# Supplementary material for: Development of a Lebanese food exchange system based on frequently consumed Eastern Mediterranean traditional dishes and Arabic sweets
Source: F1000Res. 2021 Jan 11;10:12. [Version 1] doi: 10.12688/f1000research.27461.1 (PMC7931253; doi:10.12688/f1000research.27461.1)
Supplement: Peer review history

# Peer review: Reviewer response for version 1

**DOI:** 10.5256/f1000research.30352.r78747

**Published:** 2021-03-03

**Authors:** Majid Mqbel Alkhalaf

**Affiliations:** 1 The National Nutrition Committee, Saudi Food and Drug Authority (SFDA), Riyadh, Saudi Arabia

This study is well written. I have few and minor comments need to be addressed below:

**Abstract:**

- The last part of the results seems to be part of the methodology. The results should be well written.

**Introduction:**

- The study aims need to be unified. The article aim is not matching with the abstract aim (the abstract aim is better).

**Methodology:**

- The authors need to clarify why they calculated the carbs rather than analyze them in laboratory as protein and fats.
- The authors also need to clarify how the food portions have been selected.

Is the work clearly and accurately presented and does it cite the current literature?

Yes

If applicable, is the statistical analysis and its interpretation appropriate?

Not applicable

Are all the source data underlying the results available to ensure full reproducibility?

Yes

Is the study design appropriate and is the work technically sound?

Yes

Are the conclusions drawn adequately supported by the results?

Yes

Are sufficient details of methods and analysis provided to allow replication by others?

Yes

Reviewer Expertise:

Nutrition, dietary and nutrition assessment methods, public and clinical nutrition

I confirm that I have read this submission and believe that I have an appropriate level of expertise to confirm that it is of an acceptable scientific standard.

# Peer review: Reviewer response for version 1

**DOI:** 10.5256/f1000research.30352.r77080

**Published:** 2021-03-03

**Authors:** Yonna Sacre

**Affiliations:** 1 Faculty of Arts and Sciences, Holy Spirit University of Kaslik, Jounieh, Lebanon

After reviewing the paper, I find the topic very original and useful since this is a need in our community to have a reference on which dietitians can rely to provide nutritional recommendations for patients in the clinical setting.

 On the other side, I have a few suggestions and questions for clarification:

- What are the 150 samples, since it was about the 65 items selected?
- I recommend adding the quantities of the ingredients used in the recipes of the meals selected in table 1, noting that the method of preparation and quantities can be different from one region to another.
- I recommend adding a more recent reference to be based on in order to explain how the 30 items were selected.

Is the work clearly and accurately presented and does it cite the current literature?

Yes

If applicable, is the statistical analysis and its interpretation appropriate?

I cannot comment. A qualified statistician is required.

Are all the source data underlying the results available to ensure full reproducibility?

Yes

Is the study design appropriate and is the work technically sound?

Yes

Are the conclusions drawn adequately supported by the results?

Yes

Are sufficient details of methods and analysis provided to allow replication by others?

Yes

Reviewer Expertise:

Nutrition, epidemiology, public health, obesity .

I confirm that I have read this submission and believe that I have an appropriate level of expertise to confirm that it is of an acceptable scientific standard.
